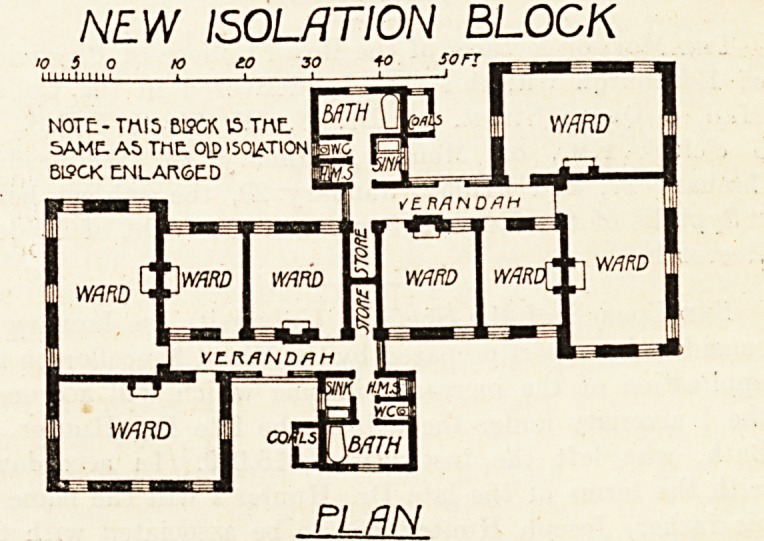# The City Hospital for Infectious Diseases, Walker Gate, Newcastle

**Published:** 1909-01-23

**Authors:** 


### THE CITY HOSPITAL FOR INFECTIOUS DISEASES, WALKER GATE, NEWCASTLE.

THIS, the first hospital for the isolation and treatment of infectious diseases to be erected by the Corporation of Newcastle, was built some twenty years ago.

As will be seen from the block plan, the scheme comprises a series of seven separate one-story ward pavilions with an administration block, laundry, and mortuary. Only five out of the seven pavilions were erected at first, giving a total accommodation of 105 beds for patients.

The recent extensions give an increase of seventy beds for patients in three entirely new pavilions and in additions to the old isolation block. Beyond this an entirely new nurses' home has been erected, the administration block two beds each, two being single-bed wards. Each group of wards is entered from a separate verandah, off which projects a sinkroom w.c., housemaid's closet, and bathroom. A small coal store entered from the open air, and a storeroom entered from the balcony complete the accommodation. For the isolation of doubtful cases or for complications the wards are admirable, but it is difficult to see how they can be efficiently administered with no sort of nurses' duty-room or ward kitchen. A block planned on the lines of the Pasteur Hospital at Paris would, we may suggest, be more economically nursed, and quite as efficient for isolation purposes.

and laundry have been enlarged, a new laboratory and dispensary, two receiving or observation blocks, a new discharge block, and a lodge for the engineer and ambulance driver have been erected.

Two of the ward pavilions are planned on the lines of the existing buildings, and contain each two wards for ten beds and two single bed wards; a ward kitchen, waiting-room, coal store, and w.c. for nurses occupy the central space between the wards, while the ward sanitary offices and bathrooms are placed in two projecting wings with cut-off lobbies at the end of each ward.

The isolation block, of which we give a detailed plan, consists of two groups of four wards, two of which contain

The old isolation block has been enlarged by the addition of two wards, each containing two beds. Two additional beds have also been obtained by the conversion of the duty-room into wards, making the total number of beds in this block six. In this case, again, it is difficult to appreciate the reason for abolishing the duty-rooms.

The two new receiving or observation blocks each contain a ward for two beds, with w.c., slop sink, and bathroom.

The discharge block contains separate dressing and undressing rooms, bathrooms, and w.c.'s for each sex.

The extensions to the administration block comprise a waiting-room and office for the matron, and similar accommodation for the resident medical officer; sitting-room, bedroom, bathroom, and w.c. for assistant resident medical officer; bedroom, bathroom, and w.c. for matron; and extensions of residents' dining-room and the kitchen offices.

The patients' laundry has been extended by the enlargement of the ironing-room and washhouse, increased drying-room accommodation, new boilers and additional machinery.

The new nurses' home is a well-planned building on three floors, with a small part four floors high, and affords accommodation for the whole of the nursing staff, with bedrooms for servants on part of the second floor and on the third floor. The one-story wing at the back contains the kitchen offices and the nurses' and servants' dining-rooms. On the ground floor of the front building are two large common rooms for nurses, and a classroom, the home sister's sitting-room, a sewing-room, seven bedrooms, a boxroom, three bathrooms (one being for the home sister), and lavatories and a cloakroom. Three staircases lead to the upper floors, and a lift is provided for luggage, coals, etc. In the bedrooms the system has been adopted of providing each room with a fireplace. In support of this arrangement, which is certainly expensive, there is this to be said, that the fireplace supplies a valuable means of ventilation, and that in the case of a nurse being ill, but not sufficiently so to be moved to a sick-room, a fireplace becomes a necessity. As a rule, however, with corridors and staircases efficiently warmed by hot-water radiators there should be no need for fires in the bedrooms.

A group of four bedrooms in the front are allocated as sick-rooms; it would surely have been better to have allotted the four end rooms, two at the front, two at the back, for this purpose, and to have provided a small pantry adjoining.

The total expenditure on building and furnishing, including the dispensary and bacteriological laboratory and the cottages for the engineer and ambulance driver, has amounted to £50,960.

The site available is now entirely covered, so that no space is left for future extension if required. If in the original plan the ward pavilions had been made two stories high instead of only one, the site could have been made to hold exactly double the number of patients. The system of one-story wards, which has been adopted in several large isolation hospitals of recent years, has nothing to justify it on any grounds; on the other hand, it is certainly more expensive in construction and wasteful in administration.

The extensions described above have been carried out from the designs of the City Architect, Mr. F. H. Holford.

## Figures and Tables

**Figure f1:**
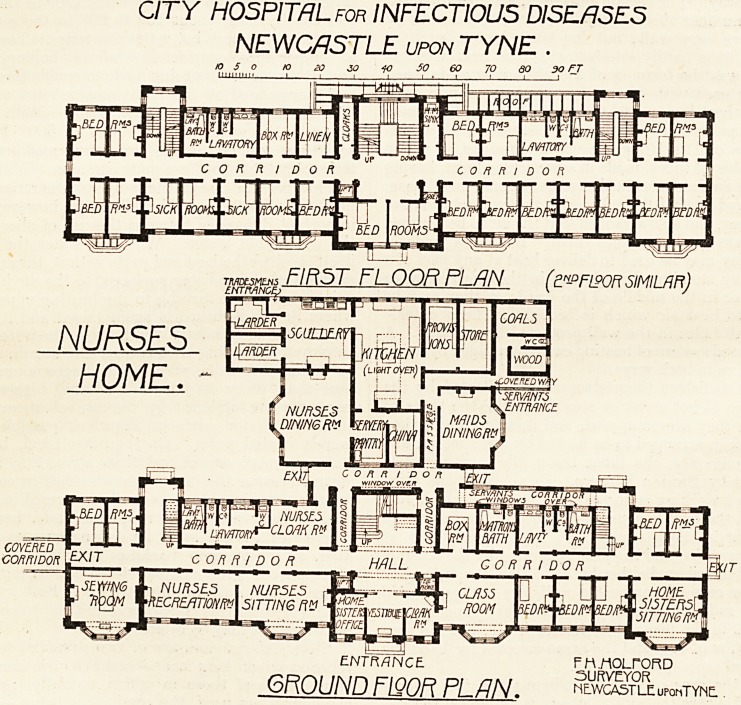


**Figure f2:**
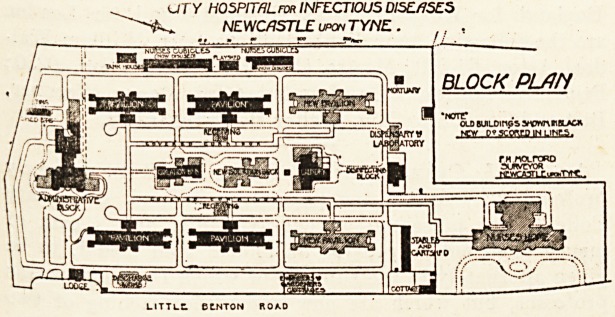


**Figure f3:**